# Supplementary material for: Smooth Interpolating Curves with Local Control and Monotone Alternating Curvature
Source: Comput Graph Forum. 2022 Oct 6;41(5):25–38. doi: 10.1111/cgf.14600 (PMC9827861; doi:10.1111/cgf.14600)
Supplement: Supplementary file 1 — Supplement Material [file CGF-41-25-s001.zip › Local-Smooth-Interpolating-MonoCurvature/extern/clothoids/submodules/quarticRootsFlocke/docs/api-cpp/file_view_hierarchy.html]

File Hierarchy — Quartic Roots v1.0.0

### Navigation

- index
- toc
- Quartic Roots »
- File Hierarchy

# File Hierarchy¶

- - File PolynomialRoots-1-Quadratic.cc
  - File PolynomialRoots-2-Cubic.cc
  - File PolynomialRoots-3-Quartic.cc
  - File PolynomialRoots-Jenkins-Traub.cc
  - File PolynomialRoots-Utils.cc
  - File PolynomialRoots-Utils.hh
  - File PolynomialRoots.hh

### Quick search

### Table of Contents

- Quartic Roots Solver
- C++ API

«
hide menu

menu
sidebar
»

### Navigation

- index
- toc
- Quartic Roots »
- File Hierarchy

© Copyright 2021, Enrico Bertolazzi.
Created using Sphinx 4.2.0.
